# Our local experience with the surgical treatment of ampullary cancer

**DOI:** 10.1186/1477-7800-2-16

**Published:** 2005-08-30

**Authors:** Dimitrios Botsios, Emmanouil Zacharakis, Ioannis Lambrou, Kostas Tsalis, Emmanouil Christoforidis, Stavros Kalfadis, Evangelos Zacharakis, Dimitrios Betsis, Ioannis Dadoukis

**Affiliations:** 1 4^th ^Surgical Department, Aristotle University of Thessaloniki, 'G. Papanikolaou' General Regional Hospital, Exohi, Thessaloniki 57010, Greece

## Abstract

**Background:**

The aim of this study is to report the outcome after surgical treatment of 32 patients with ampullary cancers from 1990 to 1999.

**Methods:**

Twenty-one of them underwent pancreaticoduodenectomy and 9 local excision of the ampullary lesion. The remaining 2 patients underwent palliative surgery.

**Results:**

When the final histological diagnosis was compared with the preoperative histological finding on biopsy, accurate diagnosis was preoperatively established in 24 patients. The hospital morbidity was 18.8% as 9 complications occurred in 6 patients. Following local excision of the ampullary cancer, the survival rate at 3 and 5 years was 77.7% and 33.3% respectively. Among the patients that underwent Whipple's procedure, the 3-year survival rate was 76.2% and the 5-year survival rate 62%.

**Conclusion:**

In this series, local resection was a safe option in patients with significant co-morbidity or small ampullary tumors less than 2 cm in size, and was associated with satisfactory long-term survival rates.

## Background

Carcinoma of the ampulla of Vater is an entity distinct from neoplasms arising from the periampullary area. Ampullary cancer accounts for some 7% of peripancreatic tumors. It is less aggressive and has a better survival than carcinomas arising from the pancreas or common bile duct [1,2]. The 5-year survival rate reported after Whipple's resection for ampullary cancer varies from 22% to 55% [3,4], whereas the relevant rate for pancreatic carcinoma is reported not to be higher than 22% to 26% [5]. Even though the outcome of patients after resection of ampullary cancer is more favorable, almost half of them will die from tumor recurrence [6]. On the other hand, pancreaticoduodenectomy has been reported to result in morbidity of 43% and mortality of 11% [7]. This fact has led to interest in local resection of ampullary tumors as described by Halstead in 1899 [8]. After local resection, it has been reported that the mortality rate reaches 7.1% and the 5-year survival rate 35% [9].

We report the outcome after surgical treatment of patients with cancer of the ampulla of Vater, with Whipple's procedure, local resection or palliative by-pass surgery.

## Methods

From 1990 to 1999, 205 patients diagnosed with periampullary neoplasms were treated in our Department. Our study population consisted of 32 of these patients that proved to have carcinoma of the ampulla of Vater, and underwent surgical treatment. They comprised 18 (56%) men and 14 (44%) women of mean age 68.5 (range: 42–77) years.

Only lesions confined to the ampulla or clearly invading the surrounding tissues from the ampulla were designated as ampullary carcinomas. Eight patients who were found histologically to have ampullary adenomas were excluded from the study as our aim was to focus on the outcome of patients with malignant lesions of the ampulla of Vater.

All the patients underwent standard diagnostic imaging investigations, which included conventional ultrasonography (US), computed tomography (CT) scan and endoscopic retrograde cholangiopancreaticography (ERCP) with multiple biopsies taken from the ampulla of Vater. Thirty (30) of the 32 patients underwent potentially radical surgery: 21 underwent pancreaticoduodenectomy (Whipple's procedure) and 9 local excision of the ampullary lesion. The remaining 2 patients underwent palliative surgery and particularly choledochojejunostomy Roux en Y. No patient received adjuvant chemotherapy or radiotherapy in the post-operative period. During the same time period, 9 patients with non-resectable ampullary cancer were not fit for operation and were treated with ERCP and stent placement. These patients that did not undergo surgical treatment were also excluded from the study. None of them received adjuvant chemotherapy or radiotherapy in the post-operative period, as well.

Pancreaticoduodenectomy was the first choice as the type of surgical treatment. Local resection was the preferable treatment when the ampullary lesion was less than 2 cm in diameter, the pre-operative biopsy showed a pT1 cancer or adenoma of the ampulla of Vater and/or the patient's concomitant medical illness or age contraindicated a major operation such as Whipple's procedure. Finally, palliative by-pass surgery was reserved for patients whose tumor was larger than 5 cm according to the findings of the preoperative imaging investigations, obstruction of the portal vein, invasion of the superior mesenteric artery and/or vein, metastatic liver disease or distant metastases.

All patients underwent regular follow-up examinations post-operatively on a 3-month basis for the first year, on a 6-month basis for the following 4 years, and annually thereafter. Follow-up included clinical examination, blood tests (CA 19-9, serum bilirubin, alkaline phosphatase), abdominal ultrasound and chest radiography.

The statistical methods employed were Fisher's exact test for comparison of proportions. Differences among groups with respect to continuous variables were tested using the Kruskal-Wallis test, whereas pairwise differences were compared by the Mann-Whitney test, at a Bonferroni-adjusted significance level. The survival curves among groups were compared with the Log-rank test and they were presented graphically with the Kaplan-Meier plots. Analyses were conducted in SPSS 11.0 (SPSS, Inc., Chicago, IL). All reported p-values are two-tailed.

## Results

All tumors were adenocarcinomas originating in the ampulla of Vater. The median size of the tumors as measured by the pathologist was 2.9 (0.8–5.1) cm. The tumor grade was recorded as well differentiated in 10, moderately differentiated in 13 and poorly differentiated in 9 patients including the two patients with unresectable tumors, according to pre-operative biopsy. Nodal involvement was microscopically found in 14 (46.6%) patients. The tumor was classified as pT1 in 8 patients and pT2-T4 in the remaining 22 patients with resectable tumors. All patients with pT1 tumors underwent local excision, while all patients with pT2-T4 tumors underwent pancreaticoduodenectomy, except one who underwent local excision.

When the final histological diagnosis after surgical treatment was compared with the preoperative histological finding on biopsy, accurate diagnosis was preoperatively established in 24 patients. In the remaining patients, the diagnosis was established intra-operatively by examination of frozen section in 3, and post-operatively by examination of the specimen in 3 patients.

The in-hospital, as well as the 30-day overall mortality rate, was 0% as no death occurred among the patients of the study. The overall hospital morbidity was 18.8%, as 9 complications occurred in 6 patients post-operatively. All the complications occurred in patients who underwent pancreaticoduodenectomy resulting in a morbidity of 28.6% in this group of patients. Although this morbidity was substantially higher compared to the group undergoing local excision and palliative surgery, this difference was not statistically significant (p = 0.129, Table 1).

**Table 1:** Comparison of different parameters among the different surgical approach groups.

	**Whipple's procedure (n = 21)**	**Local excision (n = 9)**	**Palliative surgery (n = 2)**	**p-value**
Hospital morbidity				
n (%)	6 (28.6)	0 (0)	0 (0)	0.129^a^
Hospital stay, days				
Median (range)	16 (9–32)	8 (7–10)	7.5 (7–8)	<0.001^b^

^a ^Fisher's exact test

^b ^Kruskal-Wallis test for the overall comparison (The significant pairwise comparisons with Bonferroni adjusted p-value were Whipple's procedure vs. the other two groups for both the hospital stay and operation time [p < 0.05]).

In particular, one patient presented with moderate leakage of the choledochojejunostomy, which was treated conservatively. Pancreatic fistulas were seen in two patients, which were also treated conservatively. One patient developed a sub-hepatic abscess on the 20^th ^post-operative day, but ultrasound-guided drainage was adequate treatment in this case. Surgical intervention was required in a patient who presented with intra-abdominal bleeding due to septic erosion of gastro-duodenal artery and rupture of the pancreatojejunostomy and choledochojejunostomy on the 13^th ^post-operative day. Re-operation in this case included ligation of gastro-duodenal artery, reconstruction of choledochojejunostomy and drainage of the ruptured pancreatojejunostomy. The post-operative period in this case was uneventful while the pancreatic fistula subsided within two months. Finally, two patients experienced moderate wound infection and drainage of the wound abscess was adequate treatment in these cases. On the other hand, the post-operative course was uneventful in patients who underwent either local excision or palliative operation.

Seventeen (17) patients have died during follow-up, and all but three died because of recurrence. Following palliative operation (choledochojejunostomy Roux en Y), both patients died 7 and 13 months after operation. Following local excision of the ampullary cancer in 9 patients, the survival rate at 3 years was 77.7% (7 patients) and 33.3% (3 patients) at 5 years. In the patients that achieved 5-year survival after local excision the resection was R0, the tumor was graded as pT1, N0, M0 and, moreover, it was well-differentiated.

**Figure 1:**
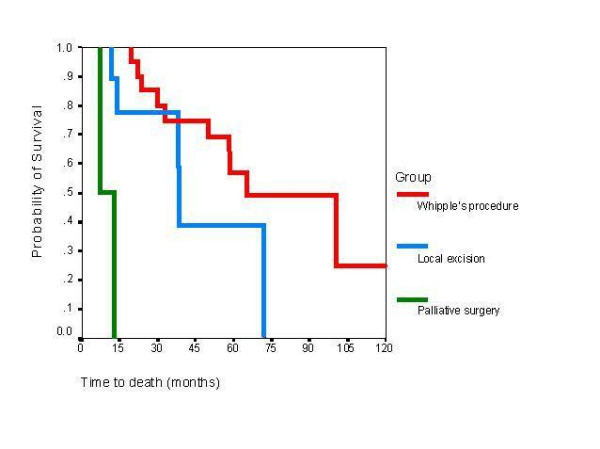
Time to death for patients who underwent pancreaticoduodenectomy, local excision and palliative surgery. Log-rank test p < 0.001.

Among 21 patients that underwent Whipple's procedure, the 3-year survival rate was 76.2% (16 patients) and the 5-year survival rate was 62% (13 patients). One patient is alive 10 years after the operation and considered cancer free. As shown in Figure 1, the median survival after Whipple's procedure was significantly higher than the median survival after local resection (65.2 vs. 38.6 months, p < 0.001).

The overall median hospital stay was 13.5 (range: 7–32) days. The median in-patient stay after Whipple's procedure was significantly greater than the median in-patient stay after local excision and palliative operation (16 vs. 8 and 7.5 days respectively, p < 0.001, Table 1).

## Discussion

Periampullary pancreatic neoplasms rank as the fifth leading cause of cancer death behind lung, breast, colorectal and prostate cancer, causing more than 30,000 deaths per year in the United States [10]. Ampullary carcinomas account for 6%–20% of all periampullary tumors and for 10.2%–36% of all operable pancreatoduodenal neoplasms [2,3,11]. They account for about 0.2% of all gastrointestinal malignancies, with a median incidence of about 57 cases per million of population per year [12,13]. In our study, cancer of the ampulla of Vater represented 15.6% of all periampullary carcinomas treated during the same time period in our Department.

A major problem in dealing with ampullary tumors is to differentiate between an adenoma and a carcinoma. Modern imaging studies such as US, CT scan, magnetic resonance imaging (MRI) and magnetic resonance cholangiopancreaticography (MRCP), have significantly improved the diagnostic accuracy in the pre-operative period. Moreover, endoscopic diagnostic techniques, such as duodenoscopy with multiple biopsies and endoscopic ultrasonography are extremely helpful tools in order to determine the nature and the extend of the tumor pre-operatively. However, despite the fact that endoscopic ultrasonography may be helpful in detecting the invasion of the tumor into the surrounding tissues and the occurrence of lymph node enlargement, it does not allow differentiation between an adenoma and a pT1 carcinoma [14]. Furthermore, duodenoscopy with multiple biopsies has been reported to miss the diagnosis of malignancy in 12% to 40% of cases [15]. Because a negative biopsy does not exclude the presence of an invasive carcinoma within the adenoma, complete excision has been recommended in all cases [16]. The above-mentioned recommendation was also the policy in our study, as we performed either Whipple's resection or local excision in all cases with a resectable ampullary tumor. In our series, duodenoscopy with biopsies failed to reveal malignancy in 20% of the patients. Endoscopic ultrasonography was not included in our diagnostic procedure due to lack of experience and relevant equipment.

Options for excision of ampullary carcinomas include local excision and pancreaticoduodenectomy. Halsted performed the first local excision for ampullary carcinoma in 1899 [8]. Although there are many case reports and a few series on the treatment of ampullary neoplasms by local ampullary resection, the criteria used to decide when local excision is suitable for certain patients are controversial, and not well addressed. For small lesions, thought to be benign pre-operatively according to endoscopic appearance and biopsy, ampullary resection is generally well accepted [17]. Bottger et al [18] stated that the indications for local excision should be that the tumor is completely removed (R0), limited to the ampulla of Vater (pT1), not poorly differentiated and with no venous/lymphatic infiltration in patients with ASA grade IV, regardless of their age. Similarly, Beger et al [19] reported that local resection is indicated in cases with pT1, N0, M0 cancer of the ampulla of Vater, excluding patients with tumor poorly differentiated. Moreover, he stated that if intra- or even post-operative histological findings show a cancer more advanced than pT1, node-positive, or poorly differentiated tumor, the procedure should be extended to a pancreaticoduodenectomy and that in patients with pT1 cancer, local excision should be always combined with a local lymph node dissection. Regarding age as an indication for local excision, we can only analyze the reported evidence about age as a contraindication for Whipple's procedure. In many authors opinion, there is rarely any justification for performing major resection in a patient over 75 years of age due to high morbidity and mortality, as well as short survival [20]. However, Kairaluoma et al [21] suggested that age is not a limiting factor for pancreatic resection and it can be performed with acceptable survival rates even in patients over 70 years of age. In general, local ampullary resection is accompanied by significantly less morbidity and mortality than pancreaticoduodenectomy [19,22]. In our study, mortality as well as morbidity was 0% among 9 patients that underwent local excision for ampullary cancer. Our criteria for performing local excision were tumor size less than 2 cm at duodenoscopy, the patient's poor medical fitness, age > 75 years and pre-operative biopsy showing a pT1 cancer or adenoma of the ampulla of Vater. In a patient less than 75 years of age, the final histological examination showed a cancer more advanced than pT1, but we did not proceed to pancreaticoduodenectomy as his comorbidity contraindicated a major operation. Finally, during local excision we performed local lymph node dissection only if the nodes were enlarged.

Pancreaticoduodenectomy is undoubtedly the procedure of choice in the management of ampullary cancer in patients who are medically fit, and this fact has been reinforced by the declining mortality after the procedure during the past decades. This was also the policy in our study; pancreaticoduodenectomy with or without pylorus preservation was the first choice of surgical treatment in patients with tumors more advanced than pT1, unless it was contraindicated by the patient's comorbidity or age (>75 years), at which point a local excision was performed. Hospital mortality after pancreaticoduodenectomy is less than 5% in recently published series, whereas the morbidity remains high and varies from 25 to 65% [6,18-20,23]. The related mortality in our series was 0% but morbidity was as high as 28.6%. The pattern of failure after surgical resection for ampullary cancer is a crucial point to assess the efficacy of adjuvant therapy for patients receiving resection for cure. No patient in our study received adjuvant chemotherapy and this is in accordance with recent published studies showing no benefit of such therapy after curative or non-curative resection of ampullary cancer [24].

Survival after local resection it is difficult to estimate given the small number of patients, but it has been reported to be 40% to 50% at 5 years [25-29]. This figure is comparable to 37.5% to 62.7% 5-year survival rate reported in the much larger pancreaticoduodenectomy series [6,18-20,23]. However, it is important to mention that the pancreaticoduodenectomy series included high-risk lesions (T3 or T4, involved nodes, poor differentiation) which are excluded from the local excision series, and this might be an explanation for the comparable survival rates. In our study which included a small number of patients, following local excision of the ampullary cancer, the survival rate at 3 and 5 years was 77.7% and 33.3% respectively. Among the patients that underwent Whipple's procedure, the 3-year survival rate was 76.2% and the 5-year survival rate 62%.

## Conclusion

In this series, local resection was a safe option in patients with significant co-morbidity or small ampullary tumors (<2 cm), and was associated with satisfactory long-term survival rates.

## Competing interests

The author(s) declare that they have no competing interests.

## Authors' contributions

DB: conceived the study, performed part of the operations and coordinated the preparation of the manuscript for submission.

EMZ: did the literature search and drafted the manuscript.

IL: participated in the design of the study, collection of data and preparation of manuscript for publication.

KT: Performed part of the operations, participated in the design of the study and preparation of manuscript for publication.

EC: Performed part of the operations, participated in the design of the study and preparation of manuscript for publication.

SK: participated in the design of the study and preparation of manuscript for publication.

EVZ: participated in the literature search and preparation of manuscript for publication and helped to draft the manuscript.

DB: has given final approval of the version to be published.

ID: has given final approval of the version to be published.
